# Stability Issues of Mycophenolic Acid in Plasma: From Patient to Laboratory

**DOI:** 10.4103/0250-474X.58193

**Published:** 2009

**Authors:** B. S. Mathew, G. T. John, S. J. Chandy, D. H. Fleming

**Affiliations:** Clinical Pharmacology Unit, Christian Medical College Hospital, Vellore-632 004, India; 1 Department of Nephrology, Christian Medical College Hospital, Vellore-632 004, India

**Keywords:** Mycophenolate, stability, transport, temperature

## Abstract

Although mycophenolate is widely prescribed in India, therapeutic drug monitoring of mycophenolic acid is not performed in most centers. This could be due to many factors such as the large investment and expertise required for high performance liquid chromatography, or the high costs involved as specialized refrigeration is required when transporting patient specimens to the laboratories with the facility to analyze MPA. The Clinical Pharmacology unit of the Christian Medical College Hospital routinely monitors the area under the curve of MPA. In order to determine if this unit could act as a central laboratory for MPA monitoring, the stability of MPA in plasma under a series of storage and transport conditions was assessed. The procedures involved the analysis of plasma specimens from patients on mycophenolate mofetil and blank plasma spiked with MPA reference standard. A range of low and high concentrations were separately analyzed to confirm long term and short term stability. The measured concentrations of MPA showed no significant change over 5 months when stored at −20° or over five days under conditions encountered during transport.

Annually approximately four thousand renal transplants are performed in a total of 100 renal transplant centres in India. Mycophenolic mofetil (Cellcept®, Roche Scientific Company (India) Pvt. Ltd., Mumbai, India) is used as a potent immunosuppressant in renal transplant patients at the Christian Medical College Hospital in India[1]. The Clinical Pharmacology unit routinely monitors the area under the curve of mycophenolic acid, the active metabolite of mofetil, by a reduced sampling strategy[2]. Although mycophenolate is widely prescribed in India, therapeutic drug monitoring of MPA is not performed in most transplant centers. Factors contributing to this include the large investment and expertise required for high performance liquid chromatography (HPLC). This may change in time. At present many hospitals wishing to monitor mycophenolic acid (MPA) will be required to send their specimens for analysis to a laboratory performing the assay. There is a reluctance to do this if specialized refrigerated transport is required due to the high cost. To determine the transport of specimens in a simple cold box containing freezer blocks is acceptable, we re-evaluated MPA stability under various conditions with the addition of stability during transport.

The parameters for bioanalytical sample stability which included long term, short term, freeze thaw, extracted sample and on instrument stability were obtained from the US FDA guidelines[3]. The stability of specimens stored at room temperature, refrigerator, freezer, in a cool box with ice packs and under conditions encountered during transport by road to the laboratory was also studied. Specimens with low (0.3-6.0 μg/ml) and high (7.0-27.4 μg/ml) concentrations were used. Blood specimens, which included either patient samples or spiked specimens were collected into EDTA-containing tubes, centrifuged, the plasma separated into clean eppendorfs, and treated as appropriate. MPA reference standard was obtained from Sigma-Aldrich, Bangalore, India.

Specimens were assayed for MPA by a previously validated HPLC assay with UV detection[2]. Two MPA stock standards were made and used separately for the MPA standard curve and as quality controls. The standards for MPA were validated and the curve was linear to 100 μg/ml with a lower limit of quantification of 0.1 μg/ml.

To measure sample preparation variability, MPA spiked specimens (5 μg/ml) were prepared on six different occasions and each analyzed against freshly prepared standards validated against known quality controls (QC's). Each of these specimens was re-analyzed within 24 h of storage at −20°. The mean measured concentration for the freshly prepared specimens was 5.02 μg/ml (range, 4.61-5.58 μg/ml; standard deviation (sd), 0.36; coefficient of variation (CV), 7%). When analyzed after 24 h the mean concentration was 4.9 μg/ml (range, 4.69-5.27; sd, 0.25; CV, 5%).

Sample extraction variability was estimated when six each of low and high concentration MPA spiked specimens were separately extracted and analyzed on the same day. The six low concentrations all measured 1.34 μg/ml and the six high had a measured mean of 11.13 μg/ml (range, 11.06-11.49; sd, 0.18). The coefficient of variation of multiple extraction analysis was nil for the low and 1.6 % for the high concentration. When the MPA extraction was performed by five different laboratory personnel, the inter-individual coefficient of variation was 2.4%. With an acceptable coefficient of variation, the stability tests were done by any of the three laboratory personnel.

Long term analyte stability was measured from three patient specimens of low (range, 1.20-3.23 μg/ml) and three of high concentrations (range, 15.64-27.4 μg/ml). They were analyzed upon collection and after one and five mo having been stored at −20°. At the time of initiation of these assays in the laboratory, MPA stability was performed over varying periods of time. Stability was indicated for chromatographic assays if periodic analysis measurements are within 15% of nominal concentrations[4]. After 1 mo, the agreement between the two readings gave a mean of 94% (range, 87-104 %) for the specimens of low concentration and 109% (range, 98-114%) for those of high concentration. After 5 mo the agreement between the two readings was a mean of 101% (range, 94-110%) for the specimens of low MPA concentration and 103% (range, 99-108%) for those of high concentration.

For freeze thaw stability, one set of specimens were re-analyzed after one freeze thaw cycle and a second set after three freeze thaw cycles. One freeze thaw cycle involved storing specimens at −20° for 24 h and then thawing on the bench-top. After specimens were completely thawed, they were refrozen for 12-24 h and the same process was repeated a total of three times[4]. Each set of specimens included three each of low (2.1, 4.23 and 5.1 μg/ml) and high (9.38, 18.8 and 30.2 μg/ml) MPA concentrations. Two of the three were patient samples and one was a sample spiked with MPA. After one freeze thaw cycle the agreement between the two readings was a mean of 101% (range, 96-113%) for the specimens of low MPA concentration and 97% (range, 95-99%) for those of high concentration. After three freeze thaw cycles the agreement between the two readings was a mean of 101% (range, 98-106%) for the specimens of low MPA concentration and 97% (range, 91-101%) for those of high concentration.

Extracted sample stability was determined by comparing both freshly extracted spiked specimens and patient's specimen of low (2.44 and 2.3 μg/ml) and high (14.8 and 23.3 μg/ml) concentrations of MPA and the same specimens after 24 h on the HPLC autoanalyser tray. The mean agreement between the two readings for the low and high concentration was 107% (range, 96-114%) and 103% (range, 100.8-104.5%), respectively.

To determine on-instrument stability, the results of spiked specimens at the beginning and re-analyzed at the end of the HPLC assay run were compared. This procedure was repeated over 6 d. On average the run time was 8 h. On 4 of the 6 d, the results were identical between the beginning and end. On the remaining 2 d the agreement was an average of 101% (range, 94-107%).

For estimation of temperature stability, low and high concentration spiked specimens were analyzed on the day of preparation. Each of the high and low spiked specimens were then aliquoted into 10 separate tubes and stored under each of the following conditions: Bench top (max/min temperature equals (32°/18°), refrigerator (4-10°), freezer (−20°) and cold box[5]. The cold boxes (20 cm square) were replicates of those to be used for the transport of MPA specimens. Each box contained two frozen ice packs of dimension 17×8 cm. Each box was sealed and only opened after the appropriate length of time. One aliquot was left on the bench top and analyzed on day 1, 2, 4 and 6. The second aliquot was left in the refrigerator and analyzed as before from day 1 to day 6. Four aliquots were stored at −20° and each analyzed on day 1, 2, 4 and 6. The last four aliquots were stored in four different cold boxes and was analyzed as mentioned above from day 1 to day 6.

Fig. 1 shows the results for the low and high spiked specimens respectively over 6 d. All results showed a change of MPA concentration less than 15% in comparison to the original concentration. Stability during transport was measured with three specimens each (2 patient samples and 1spiked) for low and high concentration. These were then stored in a standard cold box with two ice packs, as outlined above, The cold boxes were transported by road over two, three and five day following which the specimens were analyzed. The results are shown in Fig. 2.

**Fig. 1:**
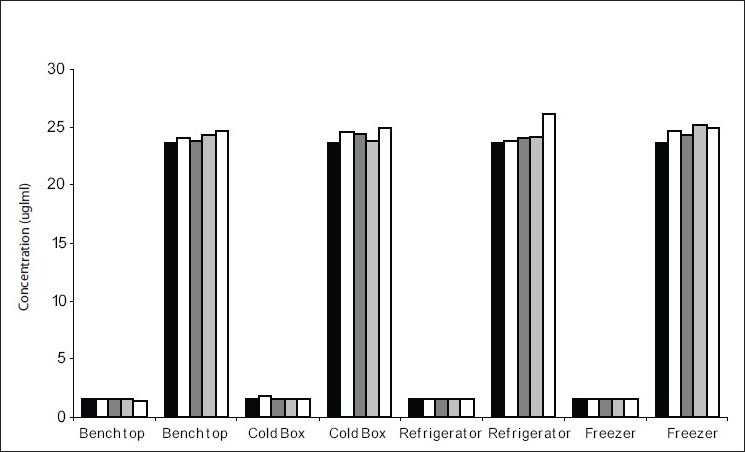
Measured concentrations under different storage conditions over six days Low (1.6 μg/ml) and high (23.6 μg/ml) concentration spiked specimens. D 0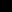, D 1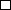, D 2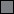, D 4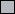 and D 6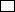.

**Fig. 2:**
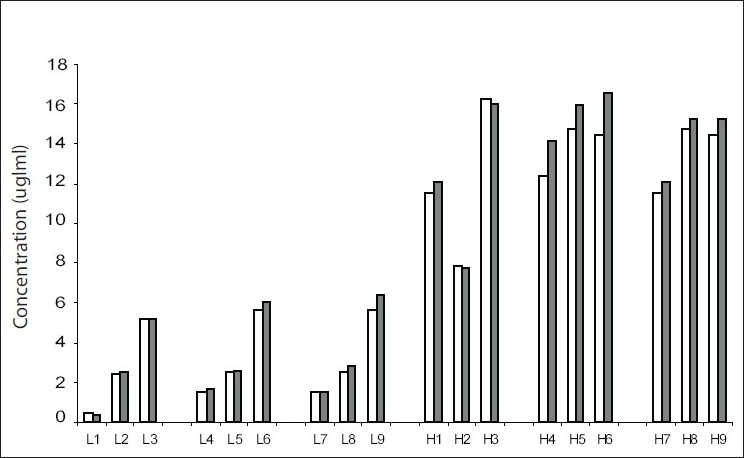
Specimens transported in a cold box over varying periods of time. L represents low concentration, while H denotes high concentration. L1, L2, L3, H1, H2, H3 for 2 days; L4, L5, L6, H4, H5, H6 for 3 days and L7, L8, L9, H7, H8, H9 for 5 days. Open bars represent analysis value on Day 0 and filled bars represent re-analysis after transport.

This fast and simple HPLC method for the measurement of MPA is highly reproducible. The various stability studies have shown that MPA is stable for up to 6 day when stored on the bench top, cold box or fridge and is stable at least up to 5 mo when stored at −20°. The stability tests have indicated that specimens can be transported for up to 5 day in a cold box with two ice packs with no loss of MPA concentration.

The cost of transporting blood specimens to metros in India with specialized refrigeration is approximately 4 times higher than transport in cold boxes (with two ice packs). A cold box with two ice packs and blood specimens (nearly ten specimens for calculation of area under the curve) would weigh less than 250 g. Transport of this from other metros would cost less than Rs 70 which is a feasible charge to an average Indian. However this stability study confirms that a cheap and easy transport facility (without specialized refrigeration) is sufficient for transport of blood specimens and will encourage hospitals to start monitoring MPA. Because of a concern of much higher temperatures in summer, we would not advocate the storage of MPA specimens at room temperature. However in the absence of a −20° freezer, MPA specimens can be stored without decay at 4° for up to 6 d. From our experience patients have benefited greatly from the monitoring of their MPA concentrations after transplant. The conclusions of this paper make the monitoring of mycophenolic assay possible for all transplant centers at minimal transport costs.
